# The Axon-Myelin Unit in Development and Degenerative Disease

**DOI:** 10.3389/fnins.2018.00467

**Published:** 2018-07-11

**Authors:** Ruth M. Stassart, Wiebke Möbius, Klaus-Armin Nave, Julia M. Edgar

**Affiliations:** ^1^Department of Neurogenetics, Max-Planck-Institute of Experimental Medicine, Göttingen, Germany; ^2^Department of Neuropathology, University Medical Center Leipzig, Leipzig, Germany; ^3^Institute of Infection, Immunity and Inflammation, College of Medical Veterinary and Life Sciences, University of Glasgow, Glasgow, United Kingdom

**Keywords:** oligodendrocyte, Schwann cell, cytoskeleton, axonal transport, energy, neuroinflammation, morphology

## Abstract

Axons are electrically excitable, cable-like neuronal processes that relay information between neurons within the nervous system and between neurons and peripheral target tissues. In the central and peripheral nervous systems, most axons over a critical diameter are enwrapped by myelin, which reduces internodal membrane capacitance and facilitates rapid conduction of electrical impulses. The spirally wrapped myelin sheath, which is an evolutionary specialisation of vertebrates, is produced by oligodendrocytes and Schwann cells; in most mammals myelination occurs during postnatal development and after axons have established connection with their targets. Myelin covers the vast majority of the axonal surface, influencing the axon's physical shape, the localisation of molecules on its membrane and the composition of the extracellular fluid (in the periaxonal space) that immerses it. Moreover, myelinating cells play a fundamental role in axonal support, at least in part by providing metabolic substrates to the underlying axon to fuel its energy requirements. The unique architecture of the myelinated axon, which is crucial to its function as a conduit over long distances, renders it particularly susceptible to injury and confers specific survival and maintenance requirements. In this review we will describe the normal morphology, ultrastructure and function of myelinated axons, and discuss how these change following disease, injury or experimental perturbation, with a particular focus on the role the myelinating cell plays in shaping and supporting the axon.

## Introduction

**Figure 1:**
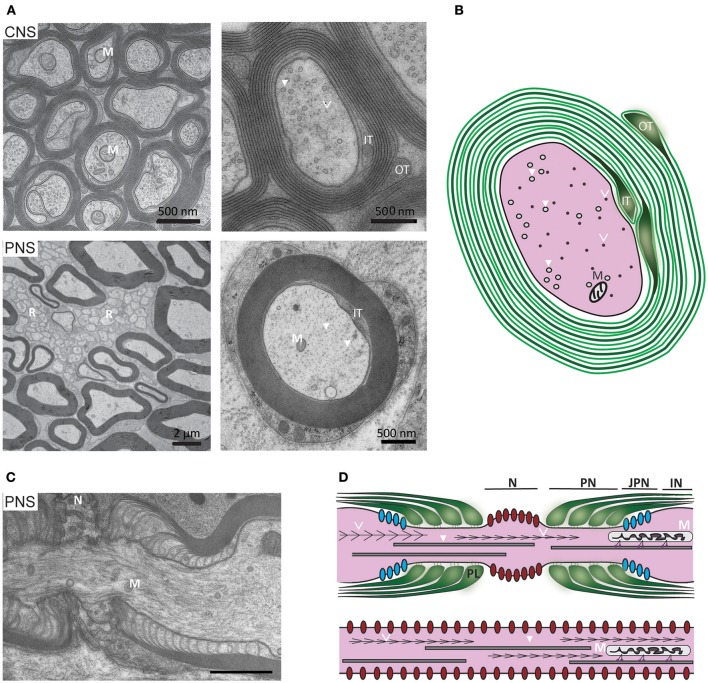
Ultrastructure of myelinated axons in the CNS and PNS. (**A**, upper) In the CNS, myelinated axons are densely packed within white matter (here: mouse optic nerve) and the myelin sheaths of neighboring fibers often directly touch. At high magnification axonal cytoskeletal elements are visible: microtubules (arrows) and neurofilaments (arrowheads). As indicated in the schematic, mitochondria are closely associated with microtubules (M, mitochondria; IT, inner tongue; OT, outer tongue). (**A**, lower) In the PNS, the Schwann cell plasma membrane is covered with a basal lamina and the myelinated fibers are separated by connective tissue. Small caliber axons are not myelinated, but organized in so-called Remak bundles (R) formed by non-myelinating Schwann cells. At high magnification, mitochondria and cytoskeletal elements can be observed. **(B)** Schematic representation of a myelinated CNS fiber: The plasma membrane of the myelinating glial cell is depicted in green. The major dense line (depicted in grey) results from the apposition of the cytoplasmic surfaces of the plasma membrane. **(C)** Electron micrograph of a PNS node of Ranvier and (**D**, upper) schematic representation of the elements of a node: paranodal loops of the myelin sheath (green), Nav1.6 channels (red), Kv1 channels (blue), neurofilaments (arrowheads), microtubules (open arrowheads), mitochondria (M), which are transported along microtubules. N, node; PN, paranode; JPN, juxta paranode; IN, internode. (**D**, lower) Schematic representation of non-myelinated axon with uniform distribution of Nav1.6 channels along the axolemma.

Neurons are highly polarized cells, with a long axon and shorter dendrites. Axons, which are the focus of this review, are unique among cellular processes, being capable of transmitting electrical impulses (spiking) and occupying, in many cases, an inordinately disproportionate amount of the neuron's volume. The axon's electrical excitability stems from the fact that **axolemma** (bolded terms are defined in Table 1) is rich in voltage-gated ion channels and ion pumps (Dumenieu et al., 2017). Extrapolation to humans, of serially reconstructed EM data from the highly branched basal cholinergic neurons of the forebrain in mice, suggests that single neurons can have a total axon length of 100 m (Wu et al., 2014). This extraordinary cellular asymmetry is initiated during early development when neuronal polarisation is established (Schelski and Bradke, 2017). All subsequent growth and maintenance of the axon is enabled by bidirectional axonal transport comprising molecular motors and microtubule tracks that convey materials between the cell body and the axon terminus. In electron micrographs, axons are easily distinguished from other cellular processes because of their unique cytoskeletal organization and, often, because they are surrounded by a myelin sheath (Figure 1).

**Table 1:** Definitions.

**Axolemma**	**The axon's plasma membrane**
Axoplasm	The axon's cytoplasm
Compact myelin	Concentric layers around the axon of double-layered oligodendroglial or Schwann cell plasma membrane, closely apposed at both intracellular and extracellular surfaces. See Figures 1A,B.
g-ratio	A measure of myelin sheath thickness, defined as the ratio of the diameter of the fiber (axon plus myelin) to the diameter of the axon alone, which is always smaller than 1. It is calculated by measuring the circumference of each on nerve cross-sections, and converting areas to diameters, assuming the circumferences are circular.
Internode	Refers to the myelinated segment of the axon; adjacent internodes are separated by a node of Ranvier. Figure 1D
Juxtaparanode	The internodal region closest to the paranode, which harbors fast voltage-gated potassium channels. Figure 1D
Node of Ranvier	Short non-myelinated axonal region that serves the generation of action potentials; morphologically a gap flanked by myelinated “internodes.” Nodes are usually ~1 μm in length and rich in voltage gated sodium channels. Figure 1D
Non-compact myelin	The part of the myelinating cell process where membranes do not compact. Consists largely of tubing around the periphery of the process as well as transient openings of previously compacted myelin in some CNS fibers, and Schmidt-Lanterman incisures in the PNS. See Figures 1, 3, 4. Also referred to as “myelinic channels.”
Paranode	The regions at either end of the internode where the non-compacted, paranodal loops of the myelin sheath about the axon Figure 1D
Septate junction	Intercellular junctions found in invertebrate epithelial cells that appear ladder-like by electron microscopy.
Spheroid	An abnormal focal swelling of the axon; mainly observed in the CNS. Often the axonal cytoskeletal elements are disorganized and axonal organelles are enriched, as observed by electron microscopy. Spheroids can also be visualized by light microscopy using respective antigen markers such as amyloid precursor protein (APP), when its transport along the axon is impaired.

## Organisation and morphology of myelinated fibres

### The ultrastructure of the myelinated axon

Recent advances in imaging modalities and in image processing have contributed enormously to our understanding of the three-dimensional composition of fibre tracts. For example, electron microscopic volume imaging, including electron tomography of fine ultrastructural details; focussed ion beam-scanning electron microscopy (FIB-SEM); and serial block-face imaging (SBF) of larger volumes, provide an increasingly complete picture of the axon-myelin unit and its neighbouring cells [see for example, Snaidero et al. (2014)].

This is not to detract from the fact that conventional transmission electron microscopy (EM) has been instrumental in elucidating the relationship between the axon and its myelin sheath as well as the subcellular components of each, in normal versus pathological or experimental conditions (reviewed in Boullerne, 2016). Nonetheless, EM requires expertise and caution in tissue preparation and image interpretation. Conventional processing of tissue samples involving chemical fixation in aqueous solution, dehydration and plastic embedding, can generate considerable artefacts; mitochondria and the lipid-rich **compact myelin** being particularly susceptible to inadequate fixation. Consequently, “changes” in either must be interpreted in relation to control material handled in parallel. Cryopreparation, combining high-pressure-freezing and freeze-substitution (HPF-FS), has become a commonplace alternative (Möbius et al., 2016) and whilst less easily obtained, cryopreparations generate useful reference samples for myelin and axon phenotypes that might otherwise be masked by artefacts of conventional preparation, in particular those caused by tissue shrinkage associated with dehydration.

Electron microscopy combined with state-of-the-art fluorescence super resolution imaging have illuminated ultrastructural details of the axonal cytoskeleton. In the adult, the axonal cytoskeleton comprises actin filaments, microtubules with microtubule associated proteins, and neurofilaments. Briefly, actin filaments, which have been observed recently in axons using fluorescence super-resolution imaging, are arranged as bundles along the axon and as periodically spaced rings underneath the axolemma, likely providing elasticity and stability, respectively (reviewed by Kevenaar and Hoogenraad, 2015; Papandreou and Leterrier, 2018). Neurofilaments and microtubules are about 100 μm long in mature axons. They are aligned with the axon's long axis and can be distinguished in electron micrographs of its cross section, being 90–100 and 230–260 Å in diameter, respectively (Peters and Vaughn, 1967; Figure 1). Neurofilament heavy, medium and light chain triplets, in combination with α-internexin in the CNS or peripherin in the PNS, fashion a semi-rigid structure, providing temporary docking sites for vesicular organelles and integrating the membrane cytoskeleton and transmembrane adhesion molecules with the axon's interior (reviewed in Kirkcaldie and Dwyer, 2017; Yuan et al., 2017). Microtubules, which are uniformly orientated with the plus-end distally (reviewed in Rao and Baas, 2018), act as scaffolds for axonal transport (Kapitein and Hoogenraad, 2011; see Axonal transport). Cross-bridging proteins, such as spectrin, bullous pemphigoid antigen, plectin and microtubule associated proteins link neurofilaments, microtubules and actin (reviewed in Yuan et al., 2017). At the **node of Ranvier** microtubules are more abundant and neurofilaments less highly phosphorylated and more closely spaced compared to the **internode**, consistent with a nodal narrowing of the axon's diameter (Figure 1) that is particularly pronounced in the PNS (Reles and Friede, 1991).

**Figure 2:**
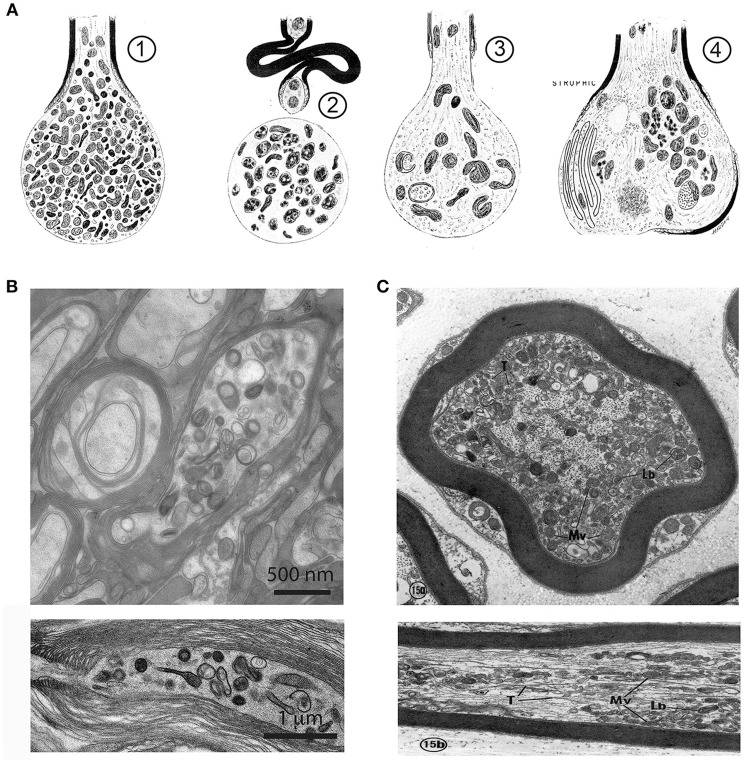
Axonal organelle accumulations after traumatic injury or interruption of axonal transport. **(A)** Early description of axonal pathology after traumatic spinal cord injury in the rat, based on electron microscopic observations. (1) After transection the axonal stump swells and accumulates mitochondria, vesicles and dense bodies. (2) In a degenerative stage the organelles disintegrate. (3) A regenerative axon is characterized by a growth cone with multiple organelles. (4) A dystrophic axonal enlargement, caused by demyelinating disease or vitamin E deficiency is characterized by abundant filaments and organelles such as mitochondria, dense bodies, layered membrane loops, and cytoplasmic dense material. Reproduced from Lampert (1967), with permission. **(B)** Electron micrographs of focal swellings and organelle accumulations in CNS axons (upper: cross section, lower: longitudinal section), here derived from white matter of adult *Plp1* null mice. Note that organelle accumulations are predominantly located in the juxtaparanodal region (lower). **(C)** Electron micrographs of axonal changes in the PNS after interruption of axonal transport in the mouse saphenous nerve by cooling. At the distal side of the transport block, retrogradely transported axonal elements accumulate along the internode. These are, among others, multivesicular bodies (Mv), lamellated bodies (Lb) which intermingle with microtubules (T), and neurofilaments (Upper: cross section, Lower: longitudinal section). Reproduced from Tsukita and Ishikawa (1980) with permission.

The **axoplasm** also contains membrane-bound organelles; mitochondria being the most obvious in electron micrographs of the healthy axon. They are typically 0.1–0.3 μm in diameter, up to 10 μm in length, and oriented parallel to the axon's long-axis. In contrast to the PNS, they are not enriched at the **node of Ranvier** or **paranode** in CNS axons (Edgar et al., 2008) where they occupy a constant fraction (1.5%) of the volume of axons >0.7 μm diameter (Perge et al., 2009), suggesting energy requirements are linearly related to axonal size. Axons also contain synaptic vesicle precursors and dense core vesicles, autophagosomes, signalling endosomes, late endosomes and lysosomes, amyloid precursor protein, BDNF vesicles, smooth endoplasmic reticulum and the machinery for localised protein synthesis (reviewed in Maday et al., 2014; Lopez-Leal et al., 2016; Spaulding and Burgess, 2017; Wu et al., 2017), which usually only become apparent following injury and regeneration (Lampert, 1967; Tsukita and Ishikawa, 1980; Court et al., 2011; Figure 2).

**Figure 3:**
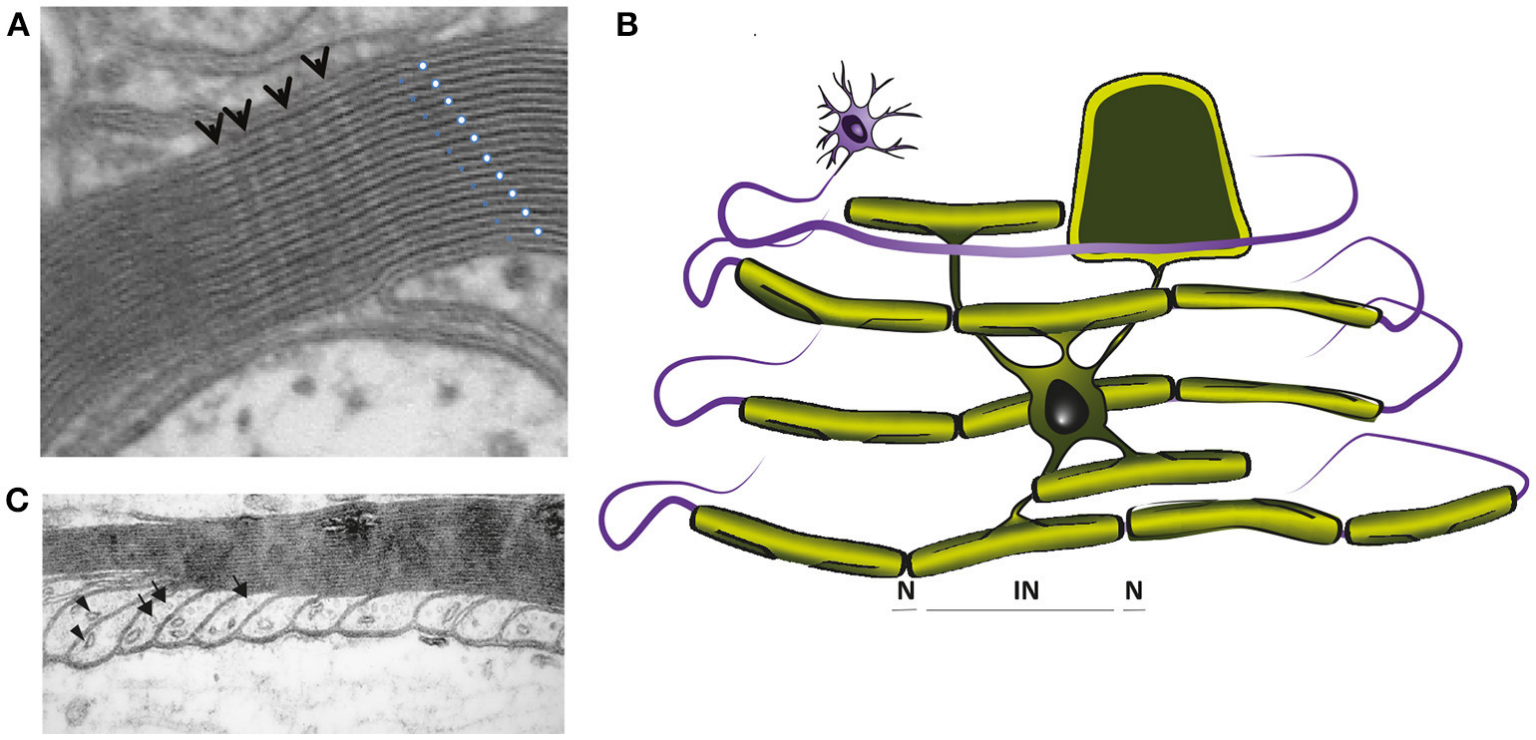
**(A)** Electron micrograph showing the multilayered myelin sheath of a CNS axon in cross section. Note the regular periodicity of compact myelin, which is composed of the major dense line, reflecting the fused intracellular glial surfaces (white dots), and the 2 nm wide intraperiod space between the extracellular surfaces of adjacent wraps (blue dots). Arrows point to radial components. **(B)** In mature CNS, a single cytoplasmic channel is present around the perimeter of the glial cell process (light green), which is best understood, if the myelin is virtually unwrapped (schematically demonstrated for one internodal segment). The cytoplasmic channel connects the glial cell soma with the most distal part of the myelin sheath and contains organelles and cytoskeletal elements, as shown in **C**. **(C)** Electron micrograph of CNS paranodal loops, abutting the axon. These contain microtubules (arrows) and vesicles (arrowheads). (**A,C** were reproduced from Edgar and Griffiths, 2013, in Diffusion MRI, From Quantitative Measurement to *in vivo* Neuroanatomy; published by Elsevier).

The myelin sheath comprises concentric wraps of the myelinating cell process around the axon, such that the lateral edges of the outermost layer abut the straddling **nodes of Ranvier**, and successively inner layers terminate increasingly distally from the node (depicted in Figures 1C,D). In **compact myelin**, an ~2 nm space (the intraperiod space) exists between the extracellular surfaces of the wrapping glial cell process whilst its intracellular surfaces are effectively fused (forming the major dense line). Together, the major dense lines and the intraperiod spaces give compact myelin its characteristic ultrastructural appearance (Figure 3A). In mature CNS myelin, a single channel of cytoplasm remains around the perimeter of the glial cell process, and live imaging of Lucifer yellow filled oligodendrocytes in *ex vivo* spinal cord slices suggests that additional transient openings are present through the compact myelin (Velumian et al., 2011). Together, these cytoplasm-filled spaces constitute the non-compact myelin or “myelinic channel” system. In the PNS, this includes Schmidt-Lanterman incisures (SLI), which connect the Schwann cell abaxonal cytoplasm with the periaxonal cytoplasm (Ghabriel and Allt, 1981).

The myelinic channel, which we suggest plays an important role in glial-mediated axonal maintenance (see Mechanisms of injury: axonal pathology caused by oligodenroglial defects), is best understood if the myelin sheath is “virtually unwrapped” (Figure 3B). It provides continuity between the cell soma and the distal-most part of the myelin sheath at the glial-axonal junction (Ransom et al., 1991), and contains septin filaments (Buser et al., 2009; Patzig et al., 2016), microtubules, vesicles (the last two illustrated in Figure 3C) and multivesicular bodies. Ion channels, glutamate receptors and metabolic transporters (e.g., monocarboxylate transporter 1) are located on its adaxonal surface (Rinholm et al., 2011; Lee et al., 2012b; Nijland et al., 2014; Domenech-Estevez et al., 2015; Saab et al., 2016). The myelin sheath is separated from the **axolemma** by the periaxonal space, an ~15 nm wide space (Peters, 1966) filled with extracellular fluid that reaches it through a spiralling pathway between the paranodal loops (reviewed in Rosenbluth, 2009). Taken together, the myelin sheath comprises a tightly wrapped double-layered cell process that is connected to its cell body via a system of fluid-filled channels.

### The myelination of axons was critical in the evolution of higher species

In the vertebrate central nervous system (CNS), myelinated axons define white matter tracts, including the corpus callosum, optic nerves, and spinal cord dorsal and ventral columns, which together account for ~40% of CNS volume in humans (Morell, 1984); a higher proportion than in other species, reflecting the fact that “connectional elaboration” (= the expansion of neuronal connectivity) was key in human brain evolution (Schoenemann et al., 2005). In the peripheral nervous system (PNS), myelinated axon bundles constitute peripheral nerves, such as the sciatic nerve and the sural nerve, which link the CNS to peripheral targets. The optic nerve, being a CNS white matter tract, is erroneously named. One of the key differences between CNS and PNS axons is that in the adult, the former do not regenerate after injury.

The evolution of a rapidly functioning, yet complex nervous system was the prerequisite for development of cognitive function in higher vertebrate species. Acceleration of nerve conduction velocity can be achieved through two basic mechanisms (Hartline and Colman, 2007a). Expansion of axonal caliber, whilst effective in invertebrates with only a few nerve fibers, such as the giant axon of the squid, and the Mauthner axon in lower vertebrates, is not viable in higher vertebrates with large number of axons and consequent space restrictions. Here, glial cells adapted to insulate the axon with a multi-lamellar, concentrically wrapped membrane, termed the myelin sheath (Hartline and Colman, 2007; Nave and Werner, 2014). In vertebrates, myelin emerged first in cartilaginous fish and is functionally homologous with, but morphologically distinct from, myelin-like glial ensheathments in annelids and crustacea, (Hartline and Colman, 2007; Zalc et al., 2008). Non-myelinating axon-ensheathing cells exist in virtually all nervous systems (Schweigreiter et al., 2006).

In adult vertebrate CNS, axons larger than the ~0.2 μm diameter “threshold” for myelination (Lee et al., 2012a; Goebbels et al., 2017) are surrounded by **compact myelin**, which is produced by oligodendrocytes. Oligodendrocyte precursors (OPCs), being present throughout life, can restore myelin sheaths following demyelination (Zawadzka et al., 2010) and potentially contribute to myelin turnover (Xiao et al., 2016). In the PNS, Schwann cells myelinate axons above a threshold size of ~1 μm diameter (Voyvodic, 1989), whilst non-myelinating Schwann cells ensheath multiple small calibre axons within so-called “Remak bundles”.

### Myelinated axons and associated cells constitute CNS white matter and PNS nerves

Within a tract or nerve, axons are packed densely and (in general) aligned in parallel. This arrangement admits the application of diffusion tensor imaging, a magnetic resonance technique used in larger brains to map fibre tracts and identify white matter changes. Axonal densities vary from one tract to another depending largely on axonal diameter and myelination status; for example, from ~70 to ~380 axons per 100 μm^2^ in cross-sections of the hippocampal commissure and basal telencephalic commissures of the adult rhesus monkey (LaMantia and Rakic, 1990). Estimates suggest that in mice, the optic nerve and corpus callosum respectively, contain ~50,000 (Edgar et al., 2010) and 300,000 (Tomasch and Macmillan, 1957) axons in total; in humans the corresponding values are ~1 and ~800 million (Koppel and Innocenti, 1983; Mikelberg et al., 1989).

Fibre tracts comprise other important cellular elements including microglia or macrophages, which provide immune surveillance in the CNS and PNS, respectively (Perry and Gordon, 1988; Klein and Martini, 2016), and astrocytes in the CNS, the processes of which often surround axons (Luse, 1956). Blood vessels, which perfuse fibre tracts, are lined by endothelial cells and associated with pericytes, smooth muscle cells and fibroblast-like cells. In humans, but not rodents, a number of neuronal cell bodies reside in the CNS white matter (Suarez-Sola et al., 2009).

### Axonal dimensions

Neurons have a single axon and these vary greatly in length as well as in ramification, as indicated before (Wu et al., 2014; Economo et al., 2016). In larger animals, axons of projection neurons can reach many meters in length (Wu et al., 2014; Wedel, 2018). This is particularly remarkable considering mammalian axons are <20 μm in diameter and their neuronal cell bodies are only some tens of microns across. In both the CNS and PNS, a range of diameters exists within and between tracts/nerves such that axons of a given length can differ in diameter ~100-fold and thus 10,000-fold by volume. Measured diameters range from <0.1 to >10 μm in the CNS and from ~0.1 to ~20 μm in the PNS, in mice (reviewed in Susuki, 2010; Edgar and Griffiths, 2013). Individual PNS nerves generally contain a wide range of axonal diameters and whilst this is also the case in the spinal cord lateral and ventral (anterior in human) columns, some CNS tracts, such as the corpus callosum, optic nerve and spinal cord dorsal (posterior in human) columns, contain predominantly small diameter axons. Physically, the most critical determinant of the calibre of a mature axon is the phosphorylation status of its medium neurofilaments (NFM; see The axonal cytoskeleton; reviewed by Kirkcaldie and Dwyer, 2017).

What is the reason for the range of diameters? Although conduction velocity increases with axonal diameter, the outcome is likely inconsequential in shorter axons and it has been speculated that information rate, which is dependent on firing rate, and consequently with axonal volume and energy use, is the crucial determinant of axonal diameter; with evolutionary pressure toward thin diameters due to space and energy constraints (Perge et al., 2012). Nonetheless, axonal diameter is not absolutely constant along the axon's length. In addition to consistent narrowing at the **node** and **paranode** (see The axonal cytoskeleton), the diameter of an individual axon fluctuates over its length (Perge et al., 2012) probably as a transient response to the movement of ions (Trigo and Smith, 2015).

### Axon initial segment

The axon initial segment (AIS) is a specialized, non-myelinated region at the very proximal end of the myelinated axon at which action potentials are initiated. At the AIS, the **axolemma** is densely populated with ion channels including Nav channels and neuronal KCNQ potassium channels (reviewed in Dumenieu et al., 2017). Thus, over its 10–60 μm length, the AIS is similar in several respects to the **node of Ranvier**, including cytoskeletal arrangements. The AIS of mature CNS neurons helps maintain neuronal polarisation by acting as a selective sieve that restricts the movement of proteins from the somatodendritic compartment into the axon (Franssen et al., 2015).

### Components of nodal/paranodal specialisations (CNS/PNS)

Distal to the AIS and at both ends of a myelin sheath (Figures 1C,D, 3B), the paranodal loops (part of the myelinic channel system) tightly appose the axon and the two are tethered by **septate**-like junctions, formed by Neurofascin 155 on the glial side and Contactin and Caspr on the axonal side (Charles et al., 2002). The node of Ranvier is the small amyelinated space between the outermost paranodal loops of adjacent myelin sheaths, whilst the juxtaparanode lies between the innermost paranodal loop and the internode proper (Figures 1C,D). In both the adult CNS and PNS, Nav1.6 Na^+^ channels are located at the **node of Ranvier** and Kv1.1, Kv1.2 K^+^ channels are localised to the **juxtaparanodal** region; although others are also present (Dumenieu et al., 2017). Various anchoring molecules including β IV spectrin and Ankyrin G as well as the paranodal-axonal transverse bands (forming **septate**-like junctions) restricts electrical activity to the nodal compartment and hinder lateral diffusion of these and other axonal membrane proteins (Rosenbluth, 2009). For detailed reviews see Rasband and Peles (2015) and Dumenieu et al. (2017). A recent study suggests that adjustment of node of Ranvier length might represent a rapid and energy-efficient mechanism for tuning the arrival time of information to its target, by altering conduction speed (Arancibia-Carcamo et al., 2017).

Although CNS and PNS nodal/paranodal structures are similar in several respects, important morphological differences include the covering of the nodal space. In the PNS, Schwann cells form the basal lamina that bridges the node, and Schwann cell microvilli contact the nodal area. In the CNS, some nodes are covered by perinodal astrocytes or OPC processes (reviewed in Rasband and Peles, 2015).

## Axo-glial signalling, axonal maintenance, and energy metabolism

### Cell autonomous and bi-directional signalling regulate axon and myelin dimensions

Distal to the AIS, the axon and the cells that myelinate it form a “symbiotic” unit. Each one uses both cell autonomous and bi-directional signaling mechanisms to initiate myelination and finely tune nodal and internodal (see Components of nodal/paranodal specialisations) dimensions to ensure the timely arrival of action potentials at the nerve terminus (Rushton, 1951; Stanford, 1987). Several recent studies have provided mechanistic insight to the molecular and cyto-architectural changes that take place in the myelinating cell during axonal engagement and wrapping, and this literature has been extensively reviewed elsewhere (Bauer et al., 2009; Snaidero and Simons, 2014; Hughes and Appel, 2016; Tricaud, 2017).

In the CNS, a neuronal “switch”, the PI3K–AKT1–mTOR pathway, may be sufficient to trigger radial axonal growth, recruitment of OPCs and progressive myelination, as suggested from the phenotype of PTEN-deficient granule cell neurons and their (ectopically) myelinated parallel fiber axons (Goebbels et al., 2017). The wrapping of the oligodendrocyte process around the axon (the final number of wraps determines the thickness of the myelin sheath) and the extension of myelin along its length (determining internodal length) are intrinsically determined by glia growth, but modulated by the diameter of the axon they wrap (Bechler et al., 2015). Recently it was suggested that axons effect precise regulation of myelin formation by dictating the targeting of mRNAs to specific subcellular locations within the myelinating cell. Indeed, localised protein synthesis in OPCs is triggered by axonal action potentials (Wake et al., 2011). Correspondingly, electrically active axons are preferentially myelinated and neural activity probably also promotes myelin plasticity in the adult (reviewed in Almeida and Lyons, 2017; Bechler et al., 2018). Thus, whereas myelination is largely driven intrinsically in oligodendrocytes (Lee et al., 2012a), it can be modulated by extrinsic factors including electrical activity of axons, acting via oligodendroglial calcium transients (Baraban et al., 2018; Krasnow et al., 2018), the glutamatergic stimulation of glucose uptake (Saab et al., 2016) and other extracellular signalling molecules (Lundgaard et al., 2013; Emery and Lu, 2015).

Notably, axonal ensheathment, radial myelin growth and myelin elongation are controlled by different mechanisms in the PNS. Prior to myelination, Schwann cells separate single axons into a stable one-to-one relationship in a process called “axonal sorting”. The established axon-myelin unit then grows in size, reaching up to 1 mm or more in internodal length (Hildebrand et al., 1994). Recently, a role for the Hippo pathway and YAP/TAZ in the integration of mechanical signals and myelination emerged in Schwann cells and has been linked to longitudinal myelin growth during development (Fernando et al., 2016; Poitelon et al., 2016). The **g-ratio**, which is a measure of the relationship between axonal diameter and myelin sheath thickness, is relatively constant for CNS and PNS axons of all diameters (Donaldson and Hoke, 1905). In the PNS, a threshold level of neuregulin on axons is required for myelin initiation and the total level of neuregulin on the axon's surface dictates the thickness of the myelin sheath (Michailov et al., 2004; Taveggia et al., 2005). Neuregulin1 downstream signaling involves PI3K/AKT and MEK/ERK pathway activation, both of which have been shown to be crucial for myelination in the PNS (Taveggia, 2016). In addition to the essential role of neuregulin1 for Schwann cell development and myelination, numerous other extrinsic and intrinsic signaling cues have been shown to regulate myelin sheath formation in the PNS, including ADAM secretases, Notch signaling and Nectin proteins (Pereira et al., 2012; Monk et al., 2015; Taveggia, 2016).

Reciprocally, oligodendrocytes and Schwann cells contribute to axonal outcomes such as the initial clustering and maintenance of sodium and potassium channels at the **node of Ranvier** and **juxtaparanode**, respectively (see Components of nodal/paranodal specialisations and Figure 1). Myelin also locally increases axonal calibre by modulating axonal neurofilament transport, as demonstrated by dynamic imaging *in vitro* (Uchida et al., 2013; Monsma et al., 2014), and phosphorylation and spacing (see The axonal cytoskeleton; Yuan et al., 2017), as evidenced by electron microscopic analysis of myelin-deficient *shiverer* mutant mice; although the responsible signalling mechanisms are not well understood. A stabilizing effect of mature myelin on the neuronal cytoskeleton is mediated in part by the myelin associated glycoprotein (MAG) which requires two axonal receptor families: sialoglycans (particularly the gangliosides GD1a and GT1b) and members of the Nogo receptor (NgR) family (Yin et al., 1998). Myelin and/or myelinating cells also influence the density of axonal mitochondria, such that non-myelinated axons and the axon initial segment (AIS) have a higher density of mitochondria than myelinated **internodes** (Bristow et al., 2002; Andrews et al., 2006).

### Axonal transport

Following the establishment of nodal/paranodal specialisations, nodal ion channels turn over slowly and are replenished by motor protein driven transport (Zhang et al., 2012). Neurons, being extremely polarised, are very dependent upon intracellular transport for the delivery and removal of proteins and organelles. ATP-dependent motor proteins utilise microtubules to transport these cargoes. Members of the extended kinesin superfamily, including kinesin-1, kinesin-2, and kinesin-3, transport their cargoes anterogradely (i.e., towards the microtubule plus-end) whilst dynein-1, in complex with its activator dynactin, is responsible for most retrograde (microtubule minus–end directed) transport. Until relatively recently, the movement of cargoes in myelinated axons *in vivo* was inferred from biochemical studies using radioactive tracers to label newly synthesised proteins, or from live imaging of cargoes in non-myelinated axons in culture, reviewed in Brauckmann (2004). Recently however, live imaging of the movement of mitochondria, peroxisomes or signalling endosomes in myelinated axons *in vivo*, has been achieved (Sorbara et al., 2014; Sleigh et al., 2017).

Classically, axonal transport is divided into “fast” and “slow” according to the bulk speeds of cargo movement. For example, vesicles and mitochondria move fast at a rate of ~1 μm s^−1^, whereas cytoskeletal components translocate slowly at speeds of ~1 mm per day; the difference being due to longer intermittent “stationery” phases of the cytoskeletal elements (Roy et al., 2000; Wang et al., 2000; Wang and Brown, 2002). Axonal transport is a highly regulated process, which is still poorly understood; motor proteins must recognise and bind specific cargoes, deliver these to the appropriate sites (e.g., the **node of Ranvier**, synapse, **internode**), change directions at axonal branch points, and offload cargo according to need. Regulation occurs, at least in part, through organelle-associated scaffolding proteins that bind to regulatory proteins, including kinases and GTPases, (reviewed in Fu and Holzbaur, 2014). Not surprisingly, transport is susceptible to perturbation following changes to motors, their regulators (Morfini et al., 2009), tracks, fuel supply and/or cargoes themselves, and manifests as organelle filled axonal swellings, neurofilament-filled swellings, thinning of the axon and/or axonal degeneration. Of note, demyelinating conditions were shown to impact anterograde axonal transport in the PNS (De Waegh et al., 1992), but the analysis of specific oligodendrocyte defects later revealed that the modulation of fast and slow transport in the CNS might be a glial function independent of myelin (Kirkpatrick et al., 2001; Edgar et al., 2004).

### Energy use and supply

Axonal transport is an energy-dependent process that contributes to the brain's total energy consumption. The human brain, which occupies ~2% of the body's mass, consumes ~20% of its resting energy production (reviewed in Engl and Attwell, 2015) and white matter is estimated to consume around one third of the energy of grey matter (Sokoloff et al., 1977). A large proportion of the white matter's energy consumption is used by neurons to pump out sodium ions using energy-dependent Na^+^-K^+^-ATPases on the internodal axolemma, (reviewed in Engl and Attwell, 2015). In axons, the turnover of actin and microtubules (Engl et al., 2017), the phosphorylation of neurofilaments and the movement of cargo-bearing motor proteins (see Axonal transport) represent additional energy-consuming functions.

Molecular motors utilise 1 molecule of ATP per 8 nm step (reviewed in Maday et al., 2014). However, dyneins are less efficient than kinesins because they take more backward steps. It has been estimated that a single vesicle traversing a 1 m long human motor neuron from neuromuscular junction back to the soma or vice versa would require a minimum of 7.5 × 10^8^ ATP or ~1.25 × 10^8^ ATP molecules, respectively (Maday et al., 2014). At an average rate of 1 μm s^−1^, this represents 7.5 × 10^2^ or 1.25 × 10^2^ ATP s^−1^. Based on 2 motile organelles per 10 μm axonal length (the maximum length of an axonal mitochondrion), 50% moving anterogradely and 50% moving retrogradely, transport would utilise ~8.75 × 10^7^ ATP s^−1^ per 1 m long axon. This equates to ~6.29 × 10^−5^ g of glucose per 24 h for a human sciatic nerve containing 1,000,000 axons. Importantly, these calculations are based on a very rough approximation of organelle numbers from our own electron microscopy images; assume only 1–2 motors simultaneously hydrolysing ATP per organelle; and no “tug-of-war” or “switch” events (Maday et al., 2014). In comparison, an estimated 1.54 × 10^9^ molecules of ATP s^−1^ per cortical neuron in the rodent brain is used in action potential generation and propagation (Attwell and Laughlin, 2001).

**Figure 4:**
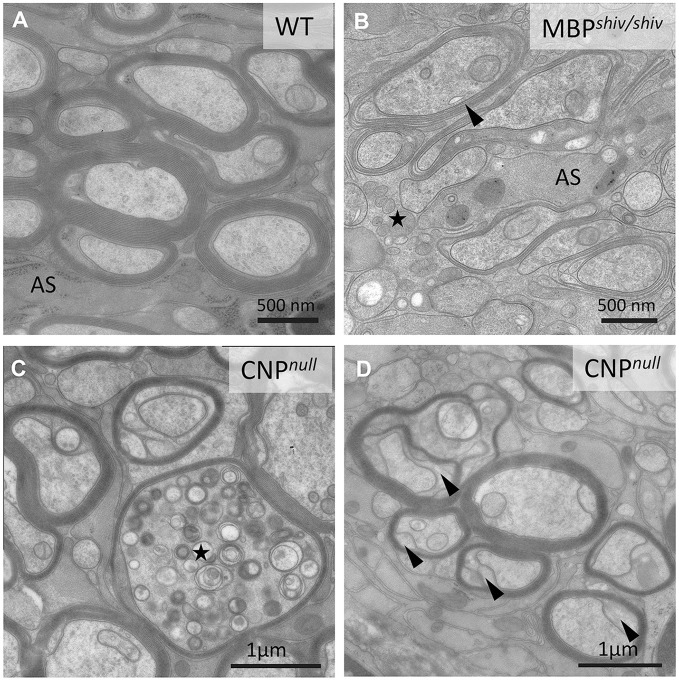
Morphological characteristics of white matter in mouse “myelin mutants”. **(A)** Normal myelinated optic nerve axons. **(B)** In the *shiverer* mouse, oligodendrocytes lacking myelin basic protein (MBP) are not able to form compact myelin, though a few loose myelin-like wraps are observed. Note the increased oligodendrocytic processes (asterisk). AS: Astrocyte process. **(C,D)** In contrast, the CNP^*null*^ mouse produces compact myelin but develops pronounced axonal swellings (asterisks in **C**) and enlarged inner tongues (arrowheads in **D**).

Whilst glucose is the main substrate for feeding the TCA cycle and for ATP production in the nervous system (Hui et al., 2017), it has been demonstrated experimentally, *ex vivo* and *in vivo*, that pyruvate or lactate can sustain neuronal function, even over several hours of high level activity (Brown et al., 2001; Wyss et al., 2011; Trevisiol et al., 2017). What are the implications? Pyruvate and lactate are the 3-carbon products of glycolysis; lactate classically being generated from pyruvate to maintain NAD^+^, and thus sustain glycolysis when oxygen levels are low. However, some cells synthesise most of their ATP by glycolysis even when oxygen is plentiful (Vander Heiden et al., 2009), especially in situations where cell growth is involved, such as development or cancer. Nonetheless, glycolysis yields only 2 molecules of ATP per 6-carbon glucose molecule and it is thought that most mammalian cells depend additionally on oxidative phosphorylation (OXPHOS; yielding ~36 molecules of ATP per glucose) to meet their energy requirements. Indeed, neurons fail to survive if OXPHOS is prevented genetically (Fukui et al., 2007; Funfschilling et al., 2012). Therefore the observation that astrocytes and post-myelination oligodendrocytes could survive as completely “glycolytic” cells (Funfschilling et al., 2012; Supplie et al., 2017) came as a surprise; although the suggestion that astrocytes (which can store glucose as glycogen) supply lactate to neurons to support their synaptic functions metabolically and when glucose supplies are low, is well-established (reviewed in Barros and Weber, 2018). More recently we proposed that glycolytic oligodendrocytes could similarly fuel ATP synthesis in axonal mitochondria with lactate (Funfschilling et al., 2012). Indeed, the monocarboxylate transporters MCT-1 and MCT-2 (which transport monocarboxylates including pyruvate and lactate) are appropriately located on the adaxonal glial cell membrane and on the axolemma, respectively (summarised schematically in Figure 4D, Funfschilling et al., 2012), and normal levels of MCT-1 in oligodendrocytes are required to maintain axonal integrity in the CNS (Lee et al., 2012b).

Why would myelinating glia act as local fuelling stations for axonal mitochondria? Possible answers are that axons require much more energy than glia and that the barrier-like properties of the myelin sheath, which play such an important role in axonal insulation, likely hamper axonal glucose uptake from the extracellular space (Nave, 2010b).

If indeed glucose is prevented from reaching the myelinated axon, then the ATP to fuel axonal energy requirements should come from the oxidative phosphorylation of glial cell glycolysis products. However, a 2013 study in *Drosophila* larvae reported that the glycolytic enzyme glyceraldehyde-3-phosphate dehydrogenase (GAPDH) is localised to axonal transport vesicles (via a huntingtin-dependent mechanism), and that vesicular transport is supported by glycolytic ATP generation (Zala et al., 2013). Subsequently, the same authors found that a mouse forebrain fraction enriched in neuronally-derived motile vesicles was associated with 8 of 10 glycolytic enzymes and could synthesise ATP *in vitro* when appropriate substrates were added (Hinckelmann et al., 2016). These data suggest that, in some neurons, the transport of vesicles can be autonomous in terms of ATP synthesis. However, subsequent studies in *Drosophila*, using cell-specific gene silencing (Volkenhoff et al., 2015), confirmed also the principle of division of labour between neurons (requiring OXPHOS but no glycolysis) and glycolytic glial cells (requiring glycolysis but not OXPHOS) and revealed a remarkable evolutionary conservation of metabolic support.

## General pathology and mechanisms of axonal injury

The unique architectural features and metabolic requirements of myelinated fibers make their axons particularly susceptible to injury from a range of diverse causes; including physical trauma, oxygen and glucose deprivation, inflammation and the consequence of gene mutations. Axonal degeneration is also an early feature of classical neurodegenerative diseases associated with characteristic pathologies of neuronal somata, such as Alzheimer's.

Axonal injury, with or without frank axonal transection can (but does not inevitably) lead to neuronal dysfunction, depending amongst other things, on the site of injury relative to axonal branch points. Clinical signs and symptoms thus reflect both the white matter tract affected and the location of injury within the tract, as observed in spinal cord injury. Below we summarize what we have learned about general mechanisms of axonal injury and degeneration from human and animal studies, but note that the mechanisms described are not necessarily distinct. For example, dying back axonopathies can also involve Wallerian-like mechanisms (Coleman, 2005).

### Mechanisms of injury: wallerian degeneration

Wallerian degeneration (WD) was described over 150 years ago by August Waller (1850), and represents the degeneration of the axon distal to injury and the subsequent clearance of axonal debris. Classically, WD is induced by physical axotomy (nerve cut) of a fiber tract, as for example in spinal cord injury and peripheral nerve trauma, but genetic evidence shows that a Wallerian-like mechanism also occurs in response to other forms of axonal injury such as “dying back” degeneration in peripheral neuropathies, and potentially in CNS axonal dystrophies in humans, as suggested from studies in mouse models (reviewed in Coleman, 2005; Conforti et al., 2014). Notably, although the rates of WD in PNS and CNS are similar, the clearance of debris by resident microglia in the CNS can take months to years, compared with a few days in the PNS (Perry et al., 1987; and reviewed in Vargas and Barres, 2007). In the PNS, Schwann cells contribute to the removal of myelin debris via a form of selective autophagy (Gomez-Sanchez et al., 2015; Jang et al., 2016). Efficient debris removal constitutes a prerequisite for subsequent regeneration in both the PNS and CNS and the failure of axonal regeneration after injury in the CNS has been linked to the presence of myelin-associated inhibitors of axonal regrowth at the injury site (reviewed in Vargas and Barres, 2007).

WD involves the remarkable process of active subcellular self-destruction. It is tightly regulated; involving a first latent phase (around 1.5–2 days post-axotomy in rodents) in which axons remain morphologically and metabolically intact and a second phase involving final breakdown of structural proteins and axonal fragmentation (reviewed in Gerdts et al., 2016). This second step is accompanied by reactive glial changes and immune cell activation (Beuche and Friede, 1984; Scheidt and Friede, 1987). In the PNS, these changes, especially the injury-response of Schwann cells, are the prerequisite of successful repair (Arthur-Farraj et al., 2012).

The Wallerian degeneration slow (*Wlds*) mouse was discovered serendipitously ~30 years ago (Perry et al., 1990) yet, despite the plethora of studies aimed at elucidating it, the mechanism of delayed axonal degeneration is still not fully understood (reviewed in Gerdts et al., 2016). The *Wlds* mutation comprises a tandem triplication resulting in the fusion of two genes (Coleman et al., 1998), the product of which is a novel chimeric protein composed of the NAD^+^ biosynthetic enzyme nicotinamide mononucleotide adenylyltransferase (NMNAT1) and a fragment of the ubiquitin ligase, Ube4b (Mack et al., 2001). NMNAT1 activity and a short N-terminal sequence are together required for the Wallerian degeneration slow effect (Avery et al., 2009; Conforti et al., 2009); most likely the extraneous NMNAT1 compensates for another NMNAT enzyme (NMNAT2) in the distal axon. NMNAT2 traffics anterogradely from the cell body into the distal axon, thus its depletion after injury (being unable to reach the axon distal to the injury and having a short half-life) is thought to initiate axonal self-destruction (Gilley and Coleman, 2010). Of note, a genetic knockout of *Nmnat2* is embryonic lethal, however, the phenotype is rescued *in vivo* upon generation of a double knockout of *Nmnat2* and *Sarm1* (Gilley et al., 2015) resulting in a normal, healthy lifespan (Gilley et al., 2017). Together, these observations led to the hypothesis that axonal depletion of NMNAT2 activates the intracellular, pro-degenerating protein SARM1 either (i) by increasing levels of NMN (the substrate for NMNAT, Di et al., 2015, 2017) and/or (ii) by decreasing NAD^+^ in a feed-forward mechanism (Gerdts et al., 2015; Sasaki et al., 2016), initiating axonal self-destruction (Osterloh et al., 2012; Gerdts et al., 2015). Of note, SARM1 has recently shown to possess an intrinsic NAD^+^ cleavage activity, which promotes axonal NAD^+^ depletion and subsequent axonal degeneration (Essuman et al., 2017). A complex interplay with other signaling cascades such as MAPK and JNK also contribute to the initiation phase (for more extensive reviews, see Conforti et al., 2014; Gerdts et al., 2016). For instance, the DLK/MAP3K12 gene has been shown to promote axonal degeneration downstream of jun kinase signaling (Miller et al., 2009; Yang et al., 2015). Moreover, MAPK signaling has been implicated in the turnover of NMNAT2, with MAPK limiting axonal NMNAT2 levels, thereby promoting axonal breakdown (Walker et al., 2017). The transition between this early phase and the subsequent “execution phase” is thought to mark the point at which axons become committed to degenerate (Conforti et al., 2014). It is now established that increased intra-axonal calcium together with calpain (a calcium-dependent protease) activation is crucial in the execution phase, leading to, amongst other things, neurofilament proteolysis and destabilization of microtubules (Zhai et al., 2003; Conforti et al., 2014; Loreto et al., 2015; Gerdts et al., 2016). Nonetheless, inhibition of calpain activation does not fully prevent the latter, indicating that additional Ca^2+^-dependent mechanisms contribute (Conforti et al., 2014). Notably, other mechanisms and organelles are likely to be involved. Indeed, overexpression of the mitochondrial NMNAT3 isoform protects axons from Wallerian degeneration (Yahata et al., 2009), and swelling and accumulation of axonal mitochondria at the paranodes represent early axonal changes in injury and disease models (for example, see Spencer and Schaumberg, 1977a; Edgar et al., 2004, and reviewed in Groh and Martini, 2017).

### Mechanisms of injury: dying back axonopathy

Dying back axonopathy is a neuropathological feature of a heterogenous group of toxic injuries and genetic defects, and generally involves long axons of the peripheral nerves and spinal cord. It is seen, for example, in Charcot-Marie-Tooth neuropathy, hereditary spastic paraplegia, giant axonal neuropathy (GAN), adrenomyeloneuropathy (AMN) and organophosphorus compound-induced delayed neurotoxicity (OPIDN). The last **three** have not been extensively reviewed elsewhere and are described briefly in Box1–3. The geometry of the susceptible axonal populations and the limitations of histological evaluation probably contributed to the misconception that, in contrast to WD, degeneration in the dying back disorders progresses retrogradely from the axonal terminus. Rather, it is likely that degeneration appears retrogradely progressing because at the level of the whole tract (comprising many axons), it is most prevalent distally (see Figure 1 of Coleman, 2005). Indeed, early ultrastructural studies on hexacarbone induced neurotoxicity in rats, demonstrated that although a retrograde temporal spread of axonal swellings occurred, axonal degeneration was not initiated at the axon terminal nor spread centripetally along individual fibers (Spencer and Schaumberg, 1977a,b). Nevertheless, axonal degeneration in these diseases seems length-dependent, pointing to a particular vulnerability of the long axons, possibly related to metabolic dysfunction or transport defects.

Box 1 **Organophosphorus (OP) compound-induced delayed neurotoxicity.**

OPIDN is an example of a pure axonal dying back neuropathy with a chemically induced degeneration of long, large-diameter sensorimotor axons in spinal cord and peripheral nerves, resulting in sensory loss and paralysis. In humans and susceptible animals, causes include occupational and accidental exposures to OP compounds (Smith and Spalding, 1959). Indeed, “Jake leg palsy”, is attributed to consumption of tri-ortho-cresyl phosphate (TOCP)-adulterated Jamaican ginger extract during the Prohibition-era in the United States. Mechanistically, OP compounds inhibit serine esterases, including neuropathy target esterase (NTE), by organophosphorylation of the active site (Makhaeva et al., 2013). Support for a role of this enzyme and its biochemical cascade in the molecular pathogenesis of OPIDN came from identification of mutations in NTE in autosomal recessive motor neuron degeneration (NTE-MND; Rainier et al., 2008). Nonetheless, the mechanisms linking exposure to a neuropathic OP compound and the onset of OPIDN shortly after, are still poorly understood.

Box 2 **Giant axonal neuropathy.**

Giant axonal neuropathy (GAN; OMIM 256850) is an interesting example of a genetically-determined progressive axonopathy that, like OPIDN, affects both sensory and motor nerves in the PNS and CNS. GAN is a rare autosomal-recessive neurodegenerative disorder caused by mutations in the GAN gene (Bomont et al., 2000), which encodes gigaxonin, a BTB-KELCH family member. GAN is characterized histologically by large axonal swellings filled with axonal intermediate filament (IF). Using dorsal root ganglia cultures, Israeli et al. (2016) demonstrated that gigaxonin is required for ubiquitin-proteasomal degradation of neuronal intermediate filaments (IF) and that IF accumulation impairs mitochondrial movement leading to metabolic and oxidative stress, implicating axonal energy insufficiency in this disorder. Symptom onset usually occurs in childhood and most patients die in the second or third decade (reviewed in Kang et al., 2016).

Box 3 **Adrenomyeloneuropathy (AMN).**

Adrenomyeloneuropathy (AMN) is caused by mutations in the X-linked gene for adenosine triphosphate binding cassette transporter (ABCD1) (Mosser et al., 1993). This peroxisomal membrane protein is required for the import of very long-chain acyl CoA esters into peroxisomes for fatty acid β oxidation (van Roermund et al., 2008). Symptom onset usually occurs in the 3rd decade and can present with or without cerebral involvement (see below). Neurological symptoms include a progressive ataxic gait and spastic paraparesis and most probably reflect the degeneration of long spinal cord axons (Pujol et al., 2002). AMN is modelled in Abcd1 mutant mice, but the molecular mechanisms that link oligodendrocyte dysfunctions in β-oxidation to axonal degeneration remain to be established. A role of lipid-derived inflammatory mediators has been proposed (Kassmann and Nave, 2008; Ruiz et al., 2015) for both AMN and the allelic disorder X-linked adrenoleukodystrophy (X-ALD), a rapidly progressive inflammatory demyelinating disorder with onset in childhood or early adolescence (Kemp et al., 2012).

### Mechanisms of injury: focal axonal degeneration and inflammation-induced axonal dysfunction

Inflammation is a primary event in multiple sclerosis (MS), neuromyelitis optica (NMO) and Guillane-Barré syndrome (GBS; a peripheral neuropathy). GBS is characterised, based on electrophysiological assessment, as primarily “demyelinating” or “axonal” and is triggered by a wide range of preceding infections, notably Campylobacter. Recent work suggests that the acute peripheral neuropathy triggered by Zika virus infection (Cao-Lormeau et al., 2016) might also be due to a GBS-like autoimmune response (see Box 4). MS, which is the best known inflammatory demyelinating disorder of the CNS, involves progressive axonal degeneration. Indeed, axonal and/or neuronal degeneration likely account for permanent neurological disability in secondary progressive MS. The causes of axonal injury in MS are still not known, and possibly include loss of trophic/metabolic support from oligodendrocytes, direct T-cell mediated injury, and energy insufficiency caused by soluble inflammatory factors (reviewed in Franklin et al., 2012).

Box 4 **Zika virus infection.**

Zika virus is a neurotropic arbovirus of the family Flaviviridae that recently received considerable attention due to its links to microcephaly and, less sensationally, to GBS (Cao-Lormeau et al., 2016). In Ifnar1 knockout mouse spinal cord and dorsal root ganglia-derived cultures (Cumberworth et al., 2017) as well as in a patient who died of Zika-GBS (Dirlikov et al., 2018), PNS neurons and Schwann cells appear rather refractory to infection. However, infection of dorsal root ganglia neurons in Ifnar1 knockout mice in vivo was reported recently (Oh et al., 2017). Differences in the experimental studies may be due to viral strain-specific effects, although dissimilarity in glycosylation of the viral coat protein, related to the cell type in which the virus was propagated pre-administration, is an another confounding factor (Alain Kohl, personal communication). In contrast, CNS glia are highly susceptible in vitro, consistent with white matter pathology in pre-term and newborn infants with congenital Zika virus infection (Chimelli et al., 2017). Whether Zika-GBS has an autoimmune etiology (Uncini et al., 2017) or is due to a direct viral neuropathogenic mechanism remains to be determined.

Experimental evidence for inflammation-induced axonal dysfunction came from the widely used animal model of MS, “experimental autoimmune encephalomyelitis” (EAE). EAE is an induced autoimmune reaction against (usually) CNS myelin components, such as myelin oligodendrocyte glycoprotein (MOG). As a tool for understanding axonal injury in human MS, EAE models have important limitations, not least that EAE is a CD4 +ve T cell mediated pathology, whereas the aetiology of MS more likely involves CD8 +ve T cells, though it is still not known and may be heterogeneous in nature (Trapp and Nave, 2008; Stys et al., 2012).

In MOG-induced EAE, Nikic et al. (2011) demonstrated axonal changes they termed “focal axonal degeneration” (FAD). Using live imaging *in vivo*, they showed that FAD, which begins with focal axonal swelling, either progressed to axonal fragmentation or resolved. Similarly, in a mouse model of Pelizaeus Merzbacher disease (PMD), defined by a primary oligodendropathy, focal axonal swellings can occur on otherwise intact (non-transected) myelinated axons (Edgar et al., 2010; see Mechanisms of injury: axonal pathology downstream of oligodendroglial defects), raising the possibility of a temporal window for therapeutic intervention. FAD can be initiated by high levels of reactive oxygen and reactive nitrogen species (ROS and RNS) alone, implicating macrophages in its evolution in EAE, and likely also in MS, where morphologically similar changes can be observed in acute lesions (Nikic et al., 2011).

This raises the question whether axons undergoing the early pre-fragmentation stage of FAD can support electrical conduction. Certainly, nitric oxide can block neural conduction in rat spinal roots (Redford et al., 1997) and axonal depolarisation and neurological signs correlate with axonal mitochondrial dysfunction and precede demyelination, in mice with MOG EAE (Sadeghian et al., 2016), supporting the suggestion that neuroinflammation *per se* can contribute to neurological dysfunction in MS and other neuroinflammatory disorders. Mechanistically, this is likely related to axonal energy deficits, as nitric oxide and reactive oxygen species can damage mitochondrial respiratory chain complexes (reviewed in Smith and Lassmann, 2002). Indeed, a deficiency in complex IV function in axons (Mahad et al., 2009) and neuronal cell bodies (reviewed in Campbell and Mahad, 2018) in MS has been reported, although the causes and consequences in the disease context are as yet unproven.

Evidence for a role for the adaptive immune system in axonal injury originated from the analysis of mouse models of genetically determined CNS and PNS disorders with secondary immune reactions. Here, a role for T-lymphocytes in secondary axonal injury was demonstrated.by crossbreeding V(D)J recombination activation (*Rag1*) knockout mice, which lack mature T and B lymphocytes, to a model of the leukodystrophy PMD, caused by overexpression of the proteolipid *PLP1* gene (see Mechanisms of injury: axonal pathology downstream of oligodendroglial defects), in which axonal changes were reduced when lymphocytes were depleted. Similar observations were made in heterozygous myelin protein zero *(Mpz)* knockout and homozygous gap junction protein b (*Gjb1*) knockout mice; models of progressive demyelinating forms of the inherited peripheral neuropathies, Charcot Marie Tooth disease (CMT; Kobsar et al., 2003 and reviewed in Groh and Martini, 2017). Mechanistically, early localised axonal changes in these mutants seem to be exacerbated by T cells “attacking” the paranodal myelin (reviewed in Groh and Martini, 2017). Nonetheless, immune cells protect myelin and axons in *Mpz* deficient mice, a genetic model of a severe dysmyelinating peripheral neuropathy, Dejerine–Sottas syndrome (DSS; Berghoff et al., 2005). Hence, the role of the adaptive immune system seems to be dependent on disease-specific mechanisms, probably including myelin integrity and the response of the innate immune system (Berghoff et al., 2005).

### Mechanisms of injury: axonal pathology caused by oligodendroglial defects

In humans, mutations in the *PLP1* gene including null, point and duplication/triplication changes, cause allelic leukodystrophies with a broad range of clinical severity, from spastic paraplegia type 2 (SPG2) to the connatal forms of PMD. All of these diseases were first identified by and later successfully modelled in *Plp1* mutant and *Plp1* overexpressing mice (Nave and Griffiths, 2004; Gruenenfelder et al., 2011). PLP and its isoform DM20 are expressed in oligodendrocytes and located in the compact myelin. The view that the survival of myelinated CNS axons is linked to the performance of surrounding glial cells initially emerged from studies of *Plp1* knockout mouse models (Griffiths et al., 1998).

**Figure 5:**
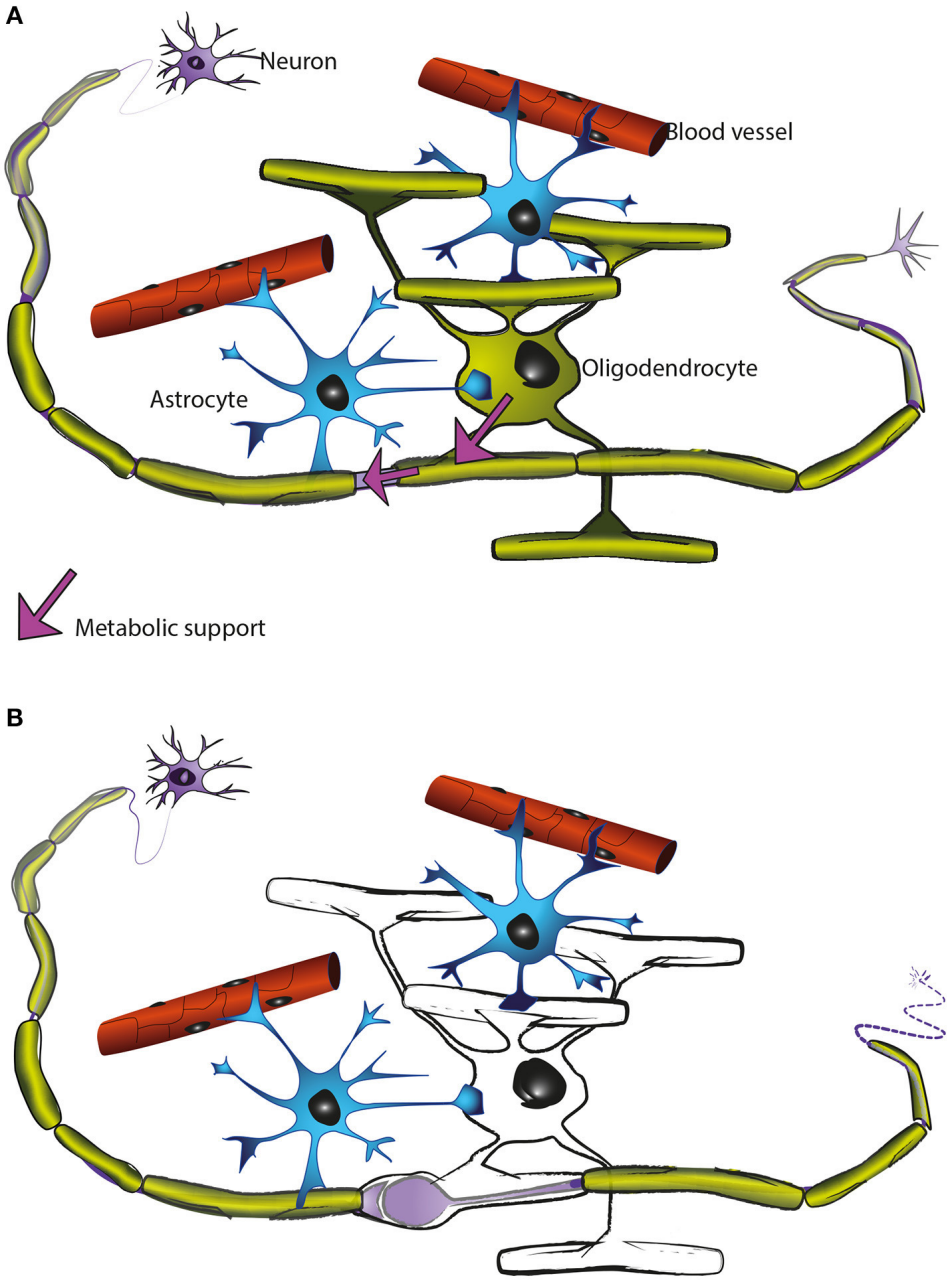
Oligodendrocytes support axons. **(A)** In the CNS, oligodendrocytes (green) provide axons with the insulating myelin sheath (green: myelinated internodes). Moreover, oligodendrocytes support axonal integrity and function independent of myelination *per se* (for details see main text). Astrocytes (blue) not only contact blood vessels but additionally interact with axons and oligodendrocytes and contribute to brain homeostasis. Oligodendrocyte precursor cells (not illustrated) are also present in the mature CNS and are the main cellular source for new oligodendrocytes after injury and in remyelination. **(B)** Oligodendrocyte dysfunction leads to a perturbed axon-glia interaction which ultimately impairs axonal health. As the myelinic channel system is likely acting as a route by which oligodendrocytes supply metabolites to the myelinated axons, any perturbation of this system could potentially impact axonal integrity, resulting for example in focal axonal swelling and distal axonal degeneration.

However, defects in myelin biosynthesis and maintenance do not inevitably lead to axonal degeneration. For example, in the spontaneously occurring Long-Evans shaker (*les*) rat and the *shiverer* mouse, which both lack a functional myelin basic protein (*Mbp)* gene (Roach et al., 1985; O'Connor et al., 1999), CNS axons are severely de- or dysmyelinated (Figures 4A,B), but degenerative axonal changes are lacking (Rosenbluth, 1980; Griffiths et al., 1998; Edgar et al., 2004; Smith et al., 2013), presumably owing to unimpaired communication between axons and engulfing oligodendroglial processes (summarised in Figure 5). Nevertheless, *shiverer* axons do have a reduced calibre and retain an “immature” cytoskeleton (Brady et al., 1999; Kirkpatrick et al., 2001). Thus, *shiverer* elucidates the profound influence the myelinating oligodendrocyte exerts on the axonal cytoskeleton and on radial axonal growth (see Cell autonomous and bi-directional signalling regulate axonal and glial dimensions). Of note, the PNS of *shiverer* mice is fully myelinated (Privat et al., 1979; Kirschner and Ganser, 1980), possibly due to overlapping functions of MBP with myelin protein zero (MPZ) and peripheral myelin protein 2 (PMP2) in peripheral myelin (Martini et al., 1995).

De- and dysmyelination are associated with the (re)distribution of sodium channels along the axolemma, thus maintaining axonal conduction (Utzschneider et al., 1993; Waxman et al., 2004). However, the resultant non-saltatory action potential propagation increases energy consumption, and accordingly, increased numbers of mitochondria have been observed in *shiverer* axons (Andrews et al., 2006) and in human MS tissue (Zambonin et al., 2011).

In contrast to *shiverer*, mice deficient in PLP or CNP (2′,3′-cyclic nucleotide 3′-phosphodiesterase) synthesise normal amounts of myelin, but develop a pronounced axonal pathology (Figures 2B, 4C,D). Taking advantage of the X-linked nature of the *Plp1* gene and female heterozygotes harbouring a mosaic of wild type and PLP-deficient myelin (the latter with subtle defects in compaction), we demonstrated that secondary axonal changes, such as organelle-filled focal swellings, are localised to the internodes formed by the PLP-deficient myelin (Edgar et al., 2004). This demonstrates that oligodendroglial support of axonal integrity is a very local function. Late onset length-dependent axonal degeneration in this mutant is likely a consequence of these early focal changes and organelle transport stasis (Edgar et al., 2004). That the axonal changes are secondary to the glial-cell defect was confirmed by cell transplantation experiments (Edgar et al., 2004) and using a novel oligodendroglia specific *Plp1* gene knockout model (Lüders et al., 2017). Axon degeneration is also the cause of progressive spasticity in the corresponding human *PLP1* null patients with SPG2 (Garbern et al., 2002); belonging to the family of disorders termed the hereditary spastic paraplegias. In mice, the absence of PLP from myelin causes a secondary loss of SIRT2, a nicotinamide adenine dinucleotide (NAD)-dependent deacetylase, which is normally abundant in myelinic channels (Werner et al., 2007). The function of SIRT2 in myelin is not understood, but as a sensor of the NAD/NADH ratio in the myelin compartment, its activity is likely regulated by oligodendrocyte glycolysis and the generation of lactate.

Similarly, *Cnp1* knockout mice (Lappe-Siefke et al., 2003), lacking a membrane-associated protein of non-compacted myelin, are fully myelinated but focal axonal swellings and degeneration of axons are observed simultaneously, commencing after postnatal day 5 (P5), in all investigated white matter tracts (Edgar et al., 2009). The encoded 2′,3′-cyclic nucleotide phosphodiesterase (CNP) most likely not serving as an enzyme, interacts with actin to antagonise MBP in myelin membrane compaction (Snaidero et al., 2017). Thus, abnormal MBP-dependent closures of the myelinic channels likely result in the observed swellings at the inner tongue, that are predicted to perturb axon-glia communication (Lappe-Siefke et al., 2003; Edgar et al., 2009; Snaidero et al., 2017).

These and other observations have led us to hypothesise that the myelinic channel system is crucial to the function of the oligodendrocyte in axonal support, likely acting as the route through which the oligodendrocyte supplies metabolites to the myelinated axon and delivers membrane proteins to the adaxonal myelin surface (Figure 5).

In the *Plp1* transgenic line #72 (Readhead et al., 1994), axonal swellings and axonal transport defects correlate with active demyelination and microglial/macrophage activation in the optic nerve. In contrast, completely demyelinated axons remain intact, suggesting injured myelin and/or neuroinflammation is relatively more detrimental than the absence of myelin *per se* (Edgar et al., 2010). Indeed, axonal injury can occur beneath “not-yet” demyelinated axons in inflammatory demyelinating models (Nikic et al., 2011; Pohl et al., 2011; Oluich et al., 2012). This supports our suggestion that axons shielded from nutrients in the extracellular milieu, are susceptible to injury if even minor damage to myelinic channels leaves oligodendrocytes unable to fuel the axon's energy requirements.

Further evidence for a role of oligodendrocytes in axonal support comes from mice lacking the *Pex5* gene (encoding the peroxisomal biogenesis factor and targeting signal type-I receptor) in myelinating glia. In this model, the absence of oligodendroglial peroxisomes does not interfere with myelination but underlies a progressive clinical phenotype caused by subcortical demyelination, inflammation and widespread axonal degeneration (Kassmann et al., 2007).

Primary oligodendrocyte death also elicits axonal changes. The diphtheria-toxin mediated ablation of oligodendrocytes in mice leads to secondary focal axonal changes and a reduction in axonal densities (Ghosh et al., 2011; Pohl et al., 2011; Oluich et al., 2012). The significant temporal delay between oligodendrocyte cell death and axonal demise can probably be explained by the initial preservation of myelin sheaths, including their content of glycolytic enzymes and metabolite transporters (Saab et al., 2016). Axonal changes in this model are independent of the adaptive immune system, as evidenced by crossbreeding to *Rag1* deficient mice (Pohl et al., 2011), however a late-onset secondary T cell response with fatal demyelination has been reported (Traka et al., 2016).

### Mechanisms of injury: axonal pathology caused by Schwann cell defects

Peripheral neuropathies are a heterogeneous group of diseases and result from inflammatory, toxic and metabolic conditions in addition to genetic defects. The last are referred to as Charcot-Marie-Tooth diseases (CMT) and can be subdivided into forms that primarily affect the Schwann cells (CMT type 1) or the axon (CMT type 2). Evidence for axonal support by Schwann cells emerged from murine mutants and transgenics for the peripheral myelin protein (*Pmp22*) gene (encoding the peripheral myelin protein of 22kDA; PMP22), which model CMT1A. A *Pmp22* point mutation defines the *Trembler* mouse (Suter et al., 1992), which is characterized by hypomyelination, demyelination, reduced axonal calibre, axonal cytoskeletal changes, and alterations in slow axonal transport (De Waegh and Brady, 1990; Kirkpatrick and Brady, 1994). Schwann cell–axon interactions are perturbed at paranodal glial-axonal junctions in *Trembler* (Robertson et al., 1997) and associated with a redistribution of axonal ion channels (Rosenbluth and Bobrowski-Khoury, 2014). Interestingly, nerve graft experiments demonstrated that axonal changes are spatially restricted to segments myelinated by “mutant” Schwann cells (De Waegh et al., 1992). Transgenic overexpression of *Pmp22* also induces dys- and demyelination and, in this case, a slowly progressive, distally pronounced, axonal loss (Sereda et al., 1996; Fledrich et al., 2014), providing a bona fide model of human Charcot-Marie-Tooth disease type 1A (CMT1A). In contrast to PMP22 overexpression in CMT1A disease, a heterozygous deletion of the *PMP22* gene causes hereditary neuropathy with liability to pressure palsies (HNPP) (Nicholson et al., 1994; Adlkofer et al., 1995; Li et al., 2007). HNPP is characterized by focal hypermyelination of peripheral nerves, and the application of mechanical compression induces a conduction block in affected patients as well as in respective animal models (Adlkofer et al., 1995; Li et al., 2007; Bai et al., 2010). Consistent with this, other CMT forms with increased myelin outfoldings, such as CMT4B, demonstrate a reduced nerve conduction velocity as well as a decreased compound muscle action potential (Previtali et al., 2007; Ng et al., 2013). However, the precise molecular mechanisms of disease progression and functional failure in CMT diseases remain only partially understood, and it is most likely that all human dysmyelinating neuropathies share a defect of Schwann cell-axon communication that is ultimately responsible for axonal conduction blocks, degeneration and a progressive clinical phenotype (Nave et al., 2007).

The role of the “mutant” glial cell versus altered myelin in PMP22-related axonal changes could not be uncoupled in these demyelinating models. Rather, it was the observation that axonal changes occur on axons with relatively (*Mag* and *Plp1* knockout mice) or completely (*Cnp1* knockout mice) normal-appearing compact myelin that provided evidence that glial cells, independent of myelin, support axonal health (Nave, 2010a; and see Mechanisms of injury: axonal pathology caused by oligodenroglial defects). Mice lacking the myelin-associated glycoprotein (*Mag*) gene, assemble CNS and PNS myelin that harbours very minor morphological changes at the inner wrap, but have reduced axon calibre associated with altered neurofilament spacing and phosphorylation in the PNS (Yin et al., 1998; Pan et al., 2005). A progressive degenerative response including paranodal myelin tomaculi and axonal loss subsequently ensues (Yin et al., 1998; Pan et al., 2005).

Just as oligodendrocytes likely provide metabolic support for axons (see Energy supply and use), so there is evidence that Schwann cells play a similar role in the PNS. Beirowski et al. (2014) demonstrated that knocking out the gene encoding the ubiquitously expressed liver kinase B1 (LKB1), specifically in adult Schwann cells, did not affect myelin morphology but led to axonal loss, most specifically of small sensory axons in Remak bundles (C fibres). LKB1 is a key regulator of energy homeostasis, suggesting axonal degeneration in this model could pertain to axonal energy insufficiency; however the phenotype is complex and its interpretation is not straightforward. More recently, using mice lacking the nutrient sensing protein O-GlcNAc transferase (OGT) in Schwann cells, the same group demonstrated that Schwann cell OGT is required for the maintenance of normal myelin and to prevent axonal loss (Kim et al., 2016). Notably, mice lacking the myelin protein Periaxin (a model for DSS), which is O-GlcNAcyated, develop a very similar phenotype (Gillespie et al., 2000; Court et al., 2004) to the conditional OGT knockout mice (Kim et al., 2016). Recently, using the compound action potential as a readout of *ex-vivo* sciatic nerve function, Rich and Brown (2018) provided evidence that non-myelinated C fibres and myelinated A fibres in the same sciatic nerve preparation, have distinct metabolic profiles. The authors showed that when fructose is supplied as the sole energy source, C fibres can utilise it directly whereas A fibres benefit through receipt of lactate from Schwann cells. Together, these data are compatible with the hypothesis that myelinating cells provide metabolic support to axons encased in compact myelin, to abrogate the consequences of their being sequestered from extracellular glucose (Nave, 2010b).

## Conclusions and implications for human diseases

In summary, we have provided an overview of the unique physical and functional properties of (myelinated) axons, with an emphasis on how these might contribute to the axon's vulnerability to injury in a variety of diseases and traumas. In particular, its relative isolation (in terms of distance) from its cell body, dependence on long-distance axonal transport, and high energy demands resulting largely from “spiking”, probably all render axons susceptible to injury from a variety of insults.

In some cases, such as SPG10, which is due to a mutation in *KIF5A* (encoding a motor protein of axonal transport), the reason why axons are vulnerable seems evident. However, the particular susceptibility of motor neuron axons in some complex disorders such as familial amyotrophic lateral sclerosis (fALS), in which the mutated gene (*SOD1*; superoxide dismutase 1) is expressed in all neural cell types, remains an enigma; although multiple mechanisms have been implicated.

In MS, a “high grade” inflammatory demyelinating disorder, axonal injury probably also entails multiple mechanisms (see Mechanisms of injury: Focal axonal degeneration and inflammation-induced axonal dysfunction) and many aspects remain poorly understood, including the direct role for lymphocytes themselves. In classical axonal and demyelinating GBS, which also fall into this category, antibody-mediated complement activation and downstream calpain-dependent proteolysis is now generally considered the most likely effector of axonal demise (Willison et al., 2016). In the “low grade” inflammatory disorder AMN, axonal degeneration happens in a length dependent fashion (Box 2), but as in fALS, the mutated *ABCD1* gene is expressed in virtually all cell types and the underlying reasons for the axonal phenotype remain poorly understood. Insight into the effectors of this and other genetically-determined length-dependent axonopathies might come from understanding how axons are injured in the neurotoxic disorders like OPIDN (Box 1). With respect to axonopathies secondary to mutations in genes whose products are expressed in oligodendrocytes and/or Schwann cells, there is increasing evidence that axonal degeneration reflects a failure of the myelinating cell to provide metabolic support to the axon. Indeed axonal energy insufficiency might represent a common pathway in multiple neurodegenerative disorders; either due to failure of metabolic support from neighbouring glia; to ischemia as in stroke; or as a consequence of failure of OXPHOS due to nitric oxide (and potentially other factors) mediated injury to axonal mitochondria (summarised in Figure 5B).

Intriguingly, in rat and mouse models of axonopathies, the obvious restriction imposed on the physical length of axons compared to humans and larger species, does not reduce their susceptibility to “length-dependent degeneration”, as for example in mouse models of SPG2 and CMT. Thus, actual physical length is not the defining factor. Rather, length-dependent degeneration in rodents is compatible with energy insufficiency being causally related to axonal demise, because presumably, energy provision is proportionally less in these small species, rendering their “long” axons just as susceptible as in larger species consuming a much greater calorie load.

Finally, axonal degeneration and/or dysfunction might not be the only axonally-related factors that contribute to neurological symptoms. Recently, we and others demonstrated reduced axonal calibre in mouse models of two complex neuropsychological disorders (Rett and Angelman syndromes; Box 5), which could potentially contribute to symptoms. Thus a variety of axonal changes, from reduced calibre, through mitochondrial dysfunction and focal swelling to transection, can probably all contribute to neurological symptoms in neurodegenerative diseases or injuries with primary or secondary involvement of axons.

Box 5 **Rett and Angelman syndromes.**

Rett and Angelman syndromes comprise part of the spectrum of neurologic disorders previously considered associated with autism (Jedele, 2007). Both present, after a short period of normal development, with global developmental delay, severe speech and communication deficits, progressive microcephaly, seizures, autistic behavior and a characteristic movement disorder. Angelman syndrome is due to loss of function of the maternally inherited ubiquitin protein ligase E3A (UBE3A) allele, while Rett syndrome is due in the majority of cases to loss of function mutation in the X-linked methyl-CpG- binding protein 2 (MECP2) gene. Remarkably, clinical features occur in the absence of evident neurodegeneration, and activation of a silenced Mecp2 allele, even with a radically truncated MeCP2 protein, in adult mice reverses neurological and morphological changes, suggesting MeCP2 might be required for maintenance of neuronal function, rather than for normal development (Guy et al., 2007; Tillotson et al., 2017). Female mice heterozygous for a Mecp2 null allele develop normally but subsequently exhibit a stiff, uncoordinated gait, tremor, breathing difficulties and hindlimb clasping. Although there is no evidence of axonal degeneration in the PNS of adult *Mecp2*^+/−^ mice, the mean diameter of sciatic nerve axons is significantly reduced and myelin is slightly thinner than normal (Bahey et al., 2017). Similarly, in a mouse model of Angelman syndrome, reduced axonal diameter and white matter abnormalities underlie impaired brain growth and microcephaly (Judson et al., 2017). Similar subtle morphological changes could, in principle, contribute to the neurological signs in Rett and Angelman syndromes.

## Author contributions

JE and RS wrote the review and provided images. JE planned and edited the review. K-AN was involved in planning, writing and finally edited the manuscript. WM contributed electron micrographs and figure legends.

### Conflict of interest statement

The authors declare that the research was conducted in the absence of any commercial or financial relationships that could be construed as a potential conflict of interest.
